# Can probiotics enhance fertility outcome? Capacity of probiotics as a single intervention to improve the feminine genital tract microbiota in non-symptomatic reproductive-aged women

**DOI:** 10.3389/fendo.2022.1081830

**Published:** 2023-01-19

**Authors:** Claudia Blancafort, Joaquín Llácer

**Affiliations:** Ginefiv Clinic, Generalife, Madrid, Spain

**Keywords:** probiotics, dysbiosis, microbiome, microbiota, fertility, implantation, pregnancy, reproductive medicine

## Abstract

Modifications in vaginal and endometrial microbiome and microbiota have been associated with fewer implantation rates and poorest pregnancy outcomes. Therefore, its study has emerged as a new biomarker in reproductive medicine. Despite the numerous papers published on probiotic use for vaginal dysbiosis and their actual wide empiric use especially for infertile patients, there is still no clear answer to justify their recommendation. The impact of probiotics on the vaginal or endometrial microbiota has often been investigated under a symptomatic altered vaginal microbial ecosystem, such as bacterial vaginosis. However 50% of women with bacterial vaginosis are asymptomatic. Actual clinical practice guidelines clearly recommend the use of specific antimicrobial agents for the management of symptomatic vaginal infections. Assuming this should be the management as well for an infertile population, what should be the treatment for the 50% non-symptomatic women presenting unfavorable vaginal/endometrial microbiota? The aim of this review is to assess the capacity of probiotics as a single intervention to alter the feminine genital tract microbiota in non-symptomatic reproductive-aged women.

## 1 Introduction

In the past two decades, great interest has emerged in the study of saprophytic microorganisms, especially since the advent of next-generation techniques based on PCR. In the gynecological field, it is well established that the female genital tract and more specifically the vagina accommodates its own microbiome, with approximately 9% of the total bacterial amount in the female body (1).

The terms microbiome and microbiota are commonly used indistinctively. However, microbiota is the collection of microorganisms coexisting in a particular site of the human body, while microbiome refers not only to microbes, but also to their genomes (2). There is a clear consensus on the fact than the vagina of healthy women of reproductive age is dominated by the *Lactobacillus* genus (3). *Lactobacillus* produces lactic acid, which then inhibits the growth of other bacteria and pathogens. When the equilibrium of this state changes, the term dysbiosis is applied, and it ranges from asymptomatic changes on the microbiome to symptomatic infectious diseases such as bacterial vaginosis (BV).

Modifications in vaginal and endometrial microbiome have been associated with gynecological consequences (4), worst reproductive outcomes (5), and infertility (6). Therefore, the study of the microbiome has emerged as a new biomarker in reproductive medicine.

When focusing on assisted reproductive technique (ART) outcomes, there seems to be a connection between abnormal vaginal microbiome and poorest pregnancy rate (7). Moreno et al. (6) have largely studied the endometrial microbiome in particular and conclude that non-*Lactobacillus* dominant microbiome (NLD, <90% of *Lactobacillus* spp. and >10% of other microbial taxa) is related to fewer implantation rates, less ongoing pregnancies, and reduced live births (8). In 2019, Koedooder et al. (9) developed a predictive model for reproductive outcome in women undergoing *in vitro* fertilization (IVF). They defined three different microbiota profiles according to which IVF success is predicted, in terms of achievable pregnancy rates (9).

A natural research question arising from this knowledge is whether vaginal and endometrial microbiome can be improved before undergoing ART by using a probiotic therapy rich in *Lactobacillus*. Despite the numerous papers published on probiotic use for vaginal dysbiosis and their actual wide empiric use, there is still no clear answer justifying their recommendation (10).

The impact of probiotics on the vaginal microbiota has often been investigated under a symptomatic altered vaginal microbial ecosystem, such as BV (11). It is known that BV affects 20%–50% of reproductive-aged women; however, 50% of women are asymptomatic (7).

Actual clinical practice guidelines clearly recommend the use of specific antimicrobial agents (antibiotic or antifungal) for the management of symptomatic vaginal infections (12). Assuming this should be the management as well for an infertile population, what should be the treatment for the 50% non-symptomatic women presenting unfavorable vaginal/endometrial microbiota? The aim of this review is to assess the capacity of probiotics as a single intervention to alter the feminine genital tract microbiota in non-symptomatic reproductive-aged women with or without infertility.

## 2 Methods

This systematic review has been elaborated following the PRISMA statement recommendations. A search of the literature published between 2002 and 2022 was performed in the PubMed database after establishing the following PICOS criteria:

Patient population or the disease being addressed: Not diseased women in reproductive age.
Interventions or exposure: Probiotics as a single treatment.
Comparator group: Placebo/absence of treatment.
Outcome or endpoint: Changes in vaginal/endometrial health parameters related to microbiota. Fertility outcomes.
Study design chosen: Clinical trials.

We searched for studies using the following terms: (microbio*” AND “fertility” AND “probiotic*”); (probiotics AND ivf); (probiotics AND embryo transfer); (“pregnancy rate” AND probiotics); (Conception AND probiotics); (“probiotics” AND infertility); (“microbio*” AND “infertility” AND “probiotic*”); (probiotic* and “endometrial microbiota”); (probiotic* and “vaginal microbiota”); (probiotic* and “vaginal microbiome”).

Article abstracts were screened, and all articles meeting the inclusion criteria were eligible for a full text assessment. References in the full text were also checked to identify further studies for inclusion.

Reasons for exclusion were as follows: articles containing no measurable endpoint on vaginal/endometrial health or fertility; publications without accessibility to the results; studies involving male partners; *in vitro* studies; pregnant and post-menopausal populations; interventions including antifungal/antibiotic treatments; studies on established diseased population (BV, vulvovaginal candidiasis, aerobic vaginitis, etc.); unpublished studies.

Information was extracted from each included trial on (i) characteristics of trial participants, (ii) method of diagnosis, (iii) type of study, (iv) site of studied microbiota, (v) size and description of the study groups, (vi) route, strain, dose, and duration of the probiotic intervention, (vii) safety, (viii) conflict of interest, (ix) objectives, and (x) outcomes.

## 3 Results

**Figure 1:**
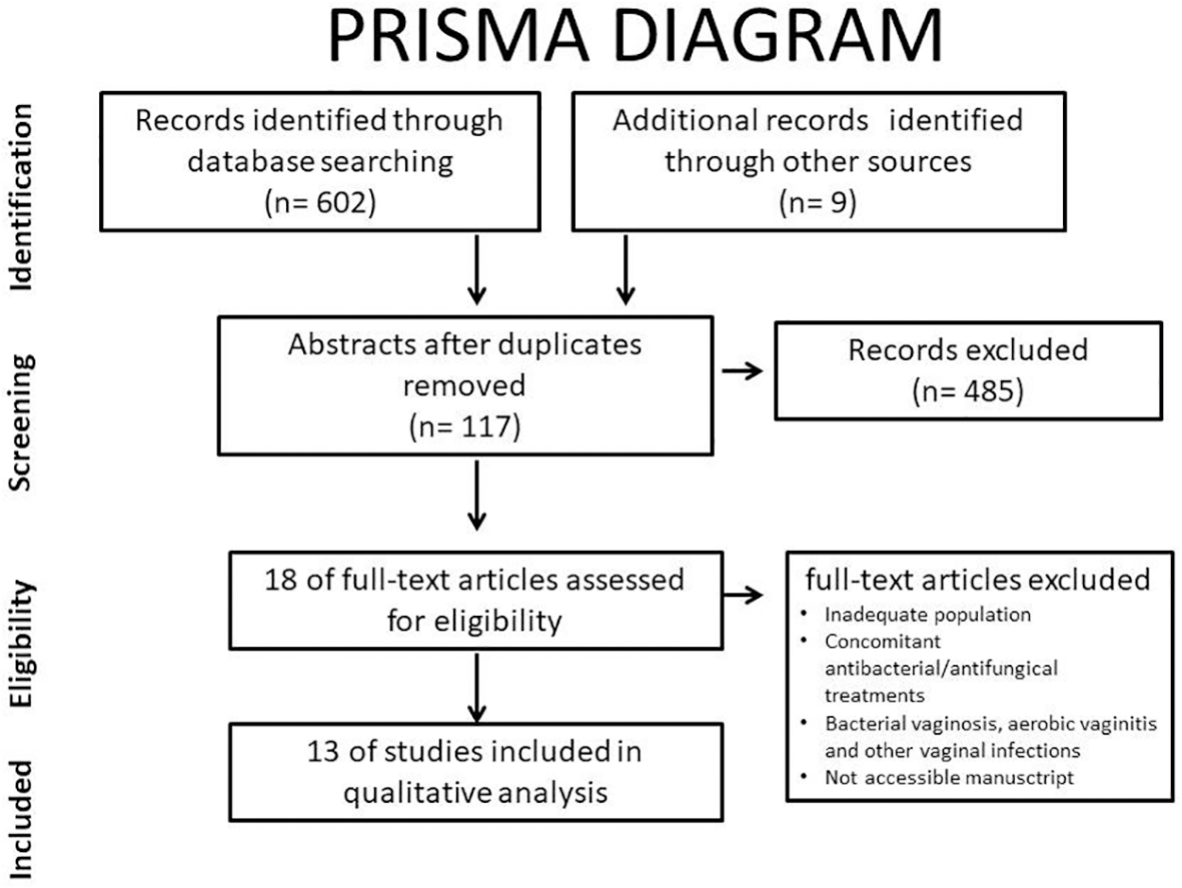
Preferred Reporting Items for Systematic Reviews and Meta-Analyses (PRISMA) diagram.

This systematic review focused on discerning whether the exclusive use of probiotics can modify the vaginal or endometrial microbiota in women of reproductive age, especially in infertile patients. After initial investigation, only 13 of the 611 papers studied were finally included in this qualitative analysis (**Figure 1**).

All 13 studies finally selected for the review were intervention studies with an intervention duration ranging between 2 days and 6 months. Eight out of 13 were randomized controlled trials with placebo and the rest were single-arm studies with no control group. The main results are presented in **Table 1**.

**Table 1:** Characteristics of included studies. NS: Nugent score.

	Disorder	Type	Size	Probiotics			Objective	Outcome/Clinical findings
				Route	Strain	Days		
13	Healthy patients	Randomized, placebo-controlled trial	64	Oral	*L. rhamnosus* and *L. fermentum*	60	To determine safety and to test induced changes in pathogen load in the vagina.	Significant increase in vaginal lactobacilli at day 28 and day 60 for lactobacilli-treated subjects versus controls.
14	Infertile patients	Randomized	117	Vaginal	*L. acidophilus*	3	To investigate the effect of probiotics on vaginal colonization and on outcome of the IVF cycle.	Treatment does not affect the vaginal colonization of *Lactobacillus* during oocyte retrieval or embryo transfer and does not improve the pregnancy rate.
15	Low lactobacilli or NS 4–6, pH >4.5	Single-arm interventional prospective study	37	Oral	*L. gasseri, L. fermentum*, and *L. plantarum*	60	To assess the degree and persistence of colonization of the administered strains and its effect on vaginal health parameters.	Colonization is diverse among participants. No subject shows colonization before 10 days. Treatment lowers pH values and improves lactobacilli counting and NS.
16	Dysbiosis, NS 4–6, low lactobacilli number pH >4.5	Multicenter, randomized, blind, placebo-controlled trial	112	Vaginal	*L. gasseri, L. fermentum*, and *L. plantarum*	7	To evaluate if the treatment could colonize and persist in the vagina and restore vaginal health parameters.	Intervention group experiences a significant reduction of vaginal pH and an increase in *L. plantarum* and *L. fermentum*. Both groups experience significant reduction of NS.
17	NS 0–6	A single-arm, pre–post study	35	Vaginal	*L. rhamnosu*s and *L. paracasei*	7	To evaluate if the treatment could colonize and persist in the vagina and restore vaginal health parameters.	50% of intermediate flora cases do not improve NS. Significant increase in the lactobacilli count but no significant changes in pH are detected.
18	NS 4–6	Double blind, randomized, placebo-controlled clinical trial	40	Oral	*L. acidophilus* and *L. rhamnosus*	15	To assess the degree and persistence of the vaginal colonization and its effect on parameters of vaginal health and subjective symptoms.	NS is significantly improved compared to placebo after 15 days of treatment. Both strains increase significantly in the vagina in comparison to baseline.
19	Self-declared healthy women	Randomized double-blind placebo-controlled crossover trial	30	Oral	*L. crispatus*	7	To evaluate safety and tolerability of the probiotic and monitor the impact on vaginal health parameters and lactobacilli counts in vagina and intestine.	Most of the changes are statistically non-significant; however, in vaginal samples, a reduction in *G. vaginalis* is noted as well as a reduction of total bacterial count. Only one of the preparations results in a significant decrease of Nugent Score.
20	NS 4–6 and pH >4.5.	Interventional, double-blind controlled clinical trial	70	Oral	*L. acidophilus, L. plantarum, fermentum*, and *L. gasseri*	60	To determine the effect of oral probiotic capsule on lactobacilli colonization and vaginal health parameters.	No significant difference in the average colonization of *Lactobacillus* or PH before and during the intervention. However, NS significantly improves.
21	Healthy women	Randomized, blind, placebo-controlled trial	23	Oral	*L. paracasei*	30	To assess the impact of probiotic on the vaginal microbiota composition and its ability to migrate to the vaginal mucosa.	The intervention does not appear to affect the stability of the vaginal CSTs; however, a significant reduction in the genus *Gardnerella* is observed.
22	Infertile women with	Prospective not randomized, not blinded	38	Oral	*L. salivarius*	180	To assess the impact on vaginal parameters between women with reproductive failure and fertile women. To evaluate the ability of *L. salivarius* to increase pregnancy rates.	Detection of lactobacilli in the vaginal samples is higher in fertile women (100%) than in women with repetitive miscarriage (57%). Oral administration of *L. salivarius* leads to a relevant number of pregnancies in infertile population together with significant changes in vaginal health parameters.
23	Infertile women without acute BV	A prospective, monocentric randomized controlled trial	80	Oral	*L. crispatus, L. rhamnosus, L. gasseri*, and *L. jensenii*	30	To investigate the effect probiotic on vaginal microbiota.	Probiotics does not influence alpha or beta diversity of the vaginal microbiota. However, a significant difference in the abundance of *U. parvum* between the probiotic group (lower) and the control group is noted.
24	Healthy women	Longitudinal study	16	Oral	*L. rhamnosus*	60	To investigate whether probiotic had any impact on the vaginal microbiome and its functional potential.	The oral probiotic has no detectable effect on either the composition or the functional potential of the vaginal microbiota.
25	NS 4–6 or pH 4.5	Pilot, open-label efficacy study	36	Oral	*L. crispatus, L. gasseri, L. acidophilus, L. rhamnosus, L. plantarum. L. brevis, L. reuteri*, and *L. paracasei*	28–48	To investigate the clinical effects on vaginal health parameters.	Significant improvement of pH was observed in all groups. No significant improvement of NS.

Only two of the finally included studies assessed the use of probiotic therapy in infertile female participants (14, 20). Fernández et al. (22) focused on women with a history of recurrent miscarriage (*n* = 21, at least three or more pregnancy losses during the first 12 weeks of pregnancy) and ART failure (*n* = 23, history of ART for at least three times, including two cycles of IVF). Patients were treated with oral *L. salivarius* for 6 months or until spontaneous pregnancy is achieved (without ART). According to their results, the intervention led to a 57% reproductive success, since 29 out of 44 treated patients got pregnant and 25 out of 44 were successful term pregnancies. Those achieving pregnancy after the intervention experienced significant changes in concentration of cultivable *Lactobacillus*, concentration of *L. salivarius*-specific DNA, pH, and Nugent Score. However, the low number of cases included and the absence of a control group using placebo within the infertile patient group are important limitations to be considered.

Despite the aforementioned inclusion criteria , none of the studies in this systematic review evaluated probiotic impact inendometrial microbiota. All the finally included studies focused on vaginalmicrobiota assessment.

Up to 11 different *Lactobacillus* strains were evaluated among the 13 studies, in solo or in different combinations. The most commonly assessed were *L. gasseri* and *L. rhamnosus* (five studies for each one), followed by *L. acidophilus*, *L. fermentum*, and *L. plantarum* (for studies for each one). Three studies evaluated *L. paracasei* and *L. crispatus*, whereas *L. salivarius*, *L. jensenii*, *L. brevis*, and *L. reuteri* were only studied in a single article.

Out of the four studies assessing *L. acidophilus*, three reported no significant changes in vaginal health parameters (14, 20, 25). Nevertheless, doses and duration of the probiotic treatments were too heterogeneous to get to solid conclusions. The other *Lactobacillus* strains presented contradictory data among the reviewed studies. Among the four studies evaluating *L. fermentum*, three reported positive outcomes in terms of vaginal presence or health parameters (13, 15, 16). Three studies precisely evaluated the combination of *L. gasseri*, *L. fermentum*, and *L. plantarum* (15, 16, 20), reaching different conclusions. The three of them evaluated *Lactobacillus* presence, pH, and Nugent Score. Tomusiak et al. (16) examined 160 women with intermediate vaginal microbiota according to Nugent Score (4–6), pH >4.5, and low or absent *Lactobacillus* count and divided them into two groups, one receiving the aforementioned vaginal probiotics and another group receiving a vaginal placebo for only 7 days. They found significant positive results in the intervention group regarding the three studied variables (16). On the other hand, Balaghi et al. (20) performed a similar study in 70 patients divided into two groups, one receiving an oral capsule of probiotics and a control group receiving placebo for 60 days. Results showed no significant differences in the Nugent Score, vaginal pH, or on *Lactobacillus* vaginal colonization (20). Strus et al. (15) also evaluated the oral delivery of the probiotics in 35 women for 60 days (with no control group). Nevertheless, they obtained contradictory results to the Balaghi et al. (20) study. They found colonization in majority of the participants after 30 days of treatment and significant reduction of pH and Nugent Score (15).

Regarding the diagnostic methods, the most commonly used were vaginal pH and Nugent Score. Only 7 out of 13 studies performed new molecular biology techniques.

Eleven out of 13 studies were conducted in European or European ascendant population; 7 out of 13 openly declared a conflict of interest, and 3 did not report such information. Only three reported total absence of disclosure.

## 4 Discussion

The debate regarding the use of probiotics in a population with asymptomatic dysbiosis before ART still has a long way to go. Although it is known that 50% of patients with dysbiosis are asymptomatic, the current insufficient evidence determines that neither empirical treatment with probiotics nor routine assessment of the vaginal/endometrial microbiota is recommended in this population. Currently, clinicians are only recommended to rule out the presence of clinical symptoms (smell, discharge, urinary tract infection, and candida) currently or during their menstrual cycle before undergoing IVF (10).

This systematic review demonstrates the difficulty in comparing studies due to the high level of heterogeneity in variables such as diagnostic method, strain used, or delivery method.

### 4.1 *Lactobacillus* strains

Assessing the possible beneficial effects of *Lactobacillus* genus on fertility seems no longer possible as a single question. Different *Lactobacillus* species have different characteristics such as the ability to produce lactic acid from degradation of glycogen conversion or capability to colonize vaginal or endometrial flora. Furthermore, their relative abundance among other species seems to play an important role too. Koedooder et al. (9) describe that a high abundance of *Lactobacillus* appears to be advantageous for IVF and IVF-ICSI outcome, but a high abundance (>60%) of specifically *L. crispatus* does not seem to be advantageous (9).

### 4.2 Delivery method

The aforementioned comparison among studies (15, 16, 20) raises the question if both delivery methods, oral or vaginal, work equally well. Tomusiak et al. (16) state that vaginally administered probiotics could have a quicker local action, driven by the activity of probiotic bacteria to colonize the vaginal epithelium, proving the efficient colonization of the abundance of *L. plantarum* and *L. fermentum* in the intervention group 100 times over versus the control group after 7 days (16). On the other hand, oral administration represents an advantage for the patient and is supposed to increase adherence to the treatment.

Most of the studies showing positive results after the treatment with *Lactobacillus* seem to agree on the fact that vaginal parameters seem to worsen once the exposition time is over. We could hypothesize then that exogen *Lactobacillus* may have difficulties in properly colonizing and replicating in the female tract epithelium.

### 4.3 Diagnostic methods

Currently, there is no gold standard in the assessment of vaginal dysbiosis (10) in the reproductive-aged population. Amsel criteria and Nugent score, the diagnostic methods classically used to detect BV, are insufficient to measure microbial complexity of the vaginal microbiota since they detect only a small number of organisms (26). Recently, the introduction of next-generation sequencing techniques has allowed complex microbiota communities to be better described, but the consensus on what threshold represents a healthy vaginal or endometrial microbiota has not yet been established.

### 4.4 Infertile population

When focusing on infertile population, only one study meets the inclusion criteria regarding the probiotic therapy as a single intervention (14). The current published data on infertile women are too heterogeneous to establish a valid diagnostic method or a treatment for vaginal dysbiosis. Two Japanese studies assessing endometrial microbiota on infertile population report pregnancy rates, but include heterogeneous concomitant antibiotic treatments for the dysbiotic profiles. Kyono et al. (27) found a higher clinical pregnancy rate in NLDM women treated with vaginal probiotics vs. no probiotics although not significant (58.8% vs. 48.0%, *p* = 0.47) (27). Kadogami et al. (28) studied 44 patients with recurrent implantation failure (RIF) and found that the therapeutic effects of the vaginal probiotic suppository seem to be higher than oral probiotics, prebiotics, and antibiotics together, except when *Gardnerella* is the main bacterium (28). In 2022, Engber (29) presents a randomized, double-blinded, placebo-controlled trial including 74 women referred for IVF. Microbiome profile is assessed using Koedooder et al.’s (9) classification (9), and they randomized patients with unfavorable vaginal microbiome to receive either vaginal probiotic capsules (containing >108 CFU of *L. gasseri* and >108 CFU of *L. rhamnosus*) or placebo. They found no significant improvement in the vaginal microbiome between the two groups but they reported a spontaneous improvement rate of 34.2% over the period of 1 to 3 months after the treatment in both groups. This would mean that expectant management of vaginal dysbiosis can also be a strategy before IVF. One of the limitations of this study mentioned by the author is the fact that the study population is Caucasian. As described in this review, very few papers study the effects of probiotics in vaginal flora of ethnicities other than Caucasian. Since microbiota seems to be significantly different between ethnicities (3), the origin of the population should also be carefully described. Maybe probiotic therapy cannot be chosen from a one-size-fits-all point of view.

Studies in infertile populations represent an even bigger challenge for the scientific community. Endometrial dysbiosis can be the cause of implantation failure and lead to infertility (8). Nevertheless, uterine microbiota can be difficult to assess since endometrial microbiota samples need to be taken invasively and the contamination risk while introducing the catheter through the vagina is high.

Control on such variables and others such as type and moment of ovarian stimulation, fresh or frozen embryo transfer, euploidy of transferred embryos, and origin of the gametes needs to be taken into account in future studies.

### 4.5 Special considerations

As described in an interesting debate article by Buggio et al. (30), one should not forget that the probiotic market has shown an important growth in the past years while the media continues to present probiotics as an appealing solution for several health issues (30).

In our review, only three of the included articles openly declared absence of conflict of interest. Only a rigorous scientific approach should guide clinicians in the decision of whether to recommend probiotic therapy, and patients should be properly informed of the current scientific evidence on the subject.

## 5 Conclusions

According to what has been previously exposed, research on the effect of probiotics on vaginal and endometrial microbiome represents an important challenge for the scientific community. *Lactobacillus*-containing vaginal probiotics hold promise for vaginal/endometrial dysbiosis and have proven to be safe, but at the moment, scientific data are insufficient to recommend their use systematically for treating asymptomatic dysbiosis before IVF. In future studies, different probiotic strains, posology, and delivery methods should be separately and meticulously evaluated in well-defined populations. Nevertheless, the first and most important step towards standardization of care should be to establish accepted boundaries of what a healthy microbiota is and by which diagnostic tools it should be assessed. Otherwise, elucidating the relationship between the probiotic treatment and fertility will remain a difficult goal to achieve. In the meantime, more conservative strategies such as expectant management could be considered.

## Author contributions

Both authors contributed equally in the review, the writing, and the submission of this paper.
